# A soft, inflatable patient support

**DOI:** 10.1308/003588413X13511609958055g

**Published:** 2013-03

**Authors:** S Shaw, M Kaushal, K Halbert

**Affiliations:** Wrightington, Wigan and Leigh NHS Foundaiton Trust, UK

## Background

Patient support is frequently required during just one part of a procedure (eg the pelvis for the perineal portion of an abdominoperineal resection of the rectum). This is commonly achieved by placing a gel patient positioner underneath the pelvis. However, this positioner can remain in place for a long time and we had noticed that patients complained occasionally of back pain after the operation. It was thought that this might be attributable to the placement of the support under the pelvis.

**Figure 1:**
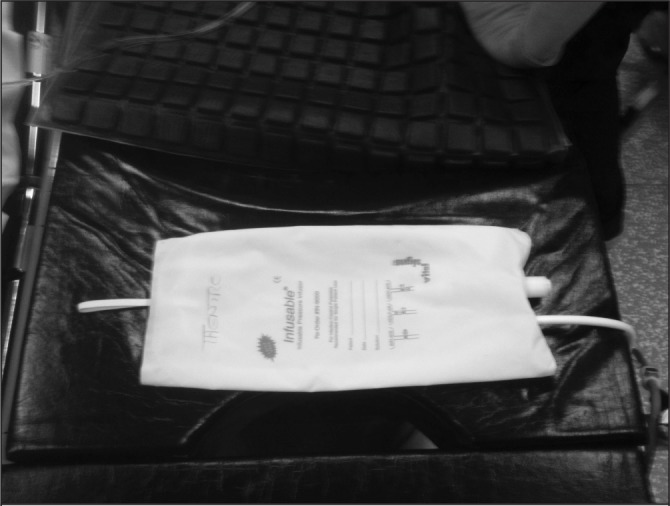
Intravenous fluid pressure bag at end of operating table

## Technique

An intravenous fluid pressure bag is placed under the pelvis at the start of the procedure. To protect the patient from direct contact with the bag and the plastic components of the device, the bag is placed under a gel pad. The pump protrudes over a lateral edge of the operating table. Having reached the point when the modified position is required, the bag is inflated for that portion of the procedure only.

**Figure 2:**
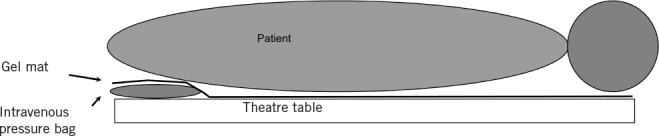
Intravenous fluid pressure bag placement

## Discussion

Since adopting this technique, no patient has complained of back pain. The technique can be applied easily to other procedures, either where a variable position is required or an unnatural position is to be avoided for the length of the procedure. Commercial inflatable patient positioners are available but we propose that this is a cheap and effective alternative. It is important that the patient is protected from the hard, plastic components of the pump mechanism.

